# Childhood Trauma and Suicide: The Mediating Effect of Stress and Sleep

**DOI:** 10.3390/ijerph19148493

**Published:** 2022-07-12

**Authors:** Ran Wu, Hong Zhu, Meng-Yang Wu, Guang-Hai Wang, Chun-Lei Jiang

**Affiliations:** 1 Counseling and Psychological Services Center, East China Normal University, Shanghai 200062, China; zhuhong@admin.ecnu.edu.cn (H.Z.); mywu@admin.ecnu.edu.cn (M.-Y.W.); 2 Department of Stress Medicine, Faculty of Psychology, Second Military Medical University, Shanghai 200433, China; 3 Pediatric Translational Medicine Institution, Shanghai Children’s Medical Center, School of Medicine, Shanghai Jiao Tong University, Shanghai 200127, China; wang-guanghai@163.com

**Keywords:** childhood trauma, childhood emotional abuse, suicide, stress, sleep, structural equation model

## Abstract

This study aimed to investigate the relationship between suicide risk, perceived stress, and sleep quality through a structural equation modeling approach. This study used convenience sampling to survey 780 undergraduate and graduate students aged 18–30 years. Students were invited to participate in the online questionnaires, which included the Beck Scale for Suicide Ideation, the Suicidal Behaviors Questionnaire-Revised, the Perceived Stress Scale, the Childhood Trauma Questionnaire-Short Form, and the Pittsburgh Sleep Quality Index. The results showed that suicide ideation and suicidal behavior were positively correlated with childhood trauma, stress, and sleep. A well-fitted structural equation model (χ^2^ = 1.52, *df* = 1, χ^2^/*df* = 1.52, *RMSEA* = 0.03, *CFI* = 1.00, *NFI* = 1.00) was constructed in this study. The hierarchical regression test showed significance in all the path coefficients of the model. The total effect of emotional abuse on suicide behaviors was 49.5%. The mediating effects accounted for 73.7% of the total effects of emotional abuse on suicidal behaviors. The results demonstrate efforts targeting stress and poor sleep might mitigate the risk of suicidal behaviors among individuals with early emotional abuse experiences.

## 1. Introduction

Globally, suicide has become a public health issue. In 2019, suicide caused more than 1 in every 100 deaths (1.3%) worldwide. Suicidal ideation and behaviors have a significant impact on individual physical and mental health, induced by the probability of suicide [1,2]. Consequently, the World Health Organization has prioritized the global reduction in suicide mortality. Thus, exploring the risk factors and causes of suicide is instrumental for suicide prevention and intervention.

The complexity and unpredictability associated with suicide risk factors have hindered suicide prevention and intervention efforts. Turecki and Brent (2016) proposed a model for evaluating suicide risk that includes both population and individual-level risk factors. Individual risk factors were grouped into distal (or predisposing) factors (e.g., family history, genetics, and early-life adversity), developmental (or mediating) factors (e.g., cognitive deficit, development of personality traits, and epigenetic factors), and proximal (or precipitating) factors (e.g., stress, sleep disturbance, and acute substance misuse). Most of these risk factors are difficult to address through psychological intervention [3].

Stress, which includes early life adversity, acute stress, and chronic stress, can increase the incidence of suicidal behavior and death by suicide [4,5]. A previous meta-analysis confirmed the association between childhood trauma and a higher risk of comorbidity with post-traumatic stress, anxiety, and attempting suicide [6]. An association between life stressors and suicide risk has also been found [7]. Furthermore, sleep disturbance was found to increase suicide risk by 1.95–2.95 times [8]. A systematic literature review showed that 70% of the reviewed studies found that at least one type of sleep problem significantly predicted suicidal ideation and behavioral outcomes among youths [9]. Stress is the physiological activity and activation of an individual’s response to the external environment. Moreover, stress can inhibit sleep and increase arousal, leading to sleep disturbances such as insomnia, nightmares, and daytime dysfunction [10]. These findings indicate that sleep may partially mediate the relationship between stress and suicide. Our team has reviewed the mechanisms linking the three variables from a neurophysiological perspective [11]. For example, the FK506-binding protein encoded by the FKBP5 gene 51 (FKBP51) not only plays an important role in regulating the hypothalamic–pituitary-adrenal axis, which regulates stress response, but also has a significant impact on sleep [12,13]. In addition, the FKBP5 gene polymorphism (rs3800373, rs9296158, rs1360780 variants) may increase suicide risks with the experience of childhood trauma [14,15]. However, no previous studies to our knowledge have examined the path from childhood trauma to suicide risk via perceived stress and sleep disturbances directly.

The development of suicide risk is a complex process. Knowing the factors associated with suicide alone is not sufficient to understand the dynamics of suicide risk. Therefore, this study aimed to enrich existing knowledge about the impact of childhood trauma on suicide risk by exploring the path of suicide risk formation. Stress and sleep disturbance were both impacted by the intervention [16]. Exploring mediators from childhood trauma to suicide risk can help us to use a variety of interventions to systematically intervene from different perspectives for cases with traumatic experiences to achieve better outcomes. In summary, we aimed to explore how childhood trauma affects suicide risk through stress and sleep to better understand the mechanisms underlying suicide. To accomplish this, we used structural equation modeling (SEM) to explore relationships between childhood trauma and suicide ideation/suicidal behaviors mediated by perceived stress and sleep quality.

## 2. Materials and Methods

### 2.1. Participants and Recruitment

This study was approved by the Committee on Ethics of Biomedicine Research, Second Military Medical University (20170305). The study was conducted in April and May 2018, during which 806 Chinese undergraduate and graduate students aged 18–30 were invited through online advertisements to participate in the online questionnaires. These students were recruited from three schools of different levels, including a key university, a general university, and a vocational college, to make our sample cover students from different schools. Of the participants, 24 did not complete all the items, and 2 participants did not meet the age requirements (i.e., they were not over 18 years old). The participants provided informed consent online before completing the questionnaire. They were also informed that participation is voluntary and that they had the right to withdraw from the study at any time. Moreover, psychological services were offered to ensure that participants felt safe and received appropriate support when required.

### 2.2. Measures

#### 2.2.1. Childhood Trauma

The Short Form of the Childhood Trauma Questionnaire (CTQ-SF) [17] is made up of 28 items (25 clinical items and 3 validity items) that retrospectively screen for a history of childhood abuse and neglect. The CTQ-SF is rated on a scale ranging from 1 (“never”) to 5 (“always”). All items are combined into five component scores: emotional abuse (EA), physical abuse (PA), sexual abuse (SA), emotional neglect (EN), and physical neglect (PN). Total scores range from 25 to 125 [18]. The cutoff scores for each of the subscales scored into moderate or severe trauma were as follows: EA ≥ 13, PA ≥ 10, SA ≥ 8, EN ≥ 15, PN ≥ 10 [19,20]. The Chinese version of the CTQ-SF used in this study has good reliability and validity among Chinese college students. The Cronbach’s alpha in this study was 0.77.

#### 2.2.2. Stress

The Perceived Stress Scale (PSS) measures perceived stress in daily life [21], and it includes 10 self-report items regarding feelings and thoughts during the past month. The PSS measures the frequency of feelings experienced by respondents that could be deemed stressful. The PSS is rated on a five-point Likert scale ranging from 0 (“never”) to 4 (“very often”), and the total score ranges from 0 to 40. The Chinese version of the PSS has been shown to have moderately high reliability (Cronbach’s alpha = 0.85) [22]. The Cronbach’s alpha in this study was 0.70.

#### 2.2.3. Sleep Quality

The Pittsburgh Sleep Quality Index (PSQI) is a questionnaire comprising 19 items that evaluate subjective sleep quality during a one-month interval [23]. All items are combined into seven component scores, with a total score ranging from 0 to 21. Higher scores indicate worse sleep quality. The PSQI has been shown to have high internal consistency, reliability, and construct validity [24]. Scores > 7 are generally used to indicate poor sleep for the Chinese version [25], and we adopted this same threshold in our study. The Cronbach’s alpha of the Chinese version of the PSQI is 0.84, and the intext has satisfactory psychometric properties [25]. The Cronbach’s alpha in this study was 0.66.

#### 2.2.4. Suicide Risk

The Beck Scale for Suicide Ideation (SSI) is a self-report questionnaire that is used to assess current suicide ideation [26]. The questionnaire consists of 19 items, with rated items ranging from 0 (“non-existent”) to 2 (“obviously existent”). Higher total scores represent more serious suicidal ideation. The SSI has good reliability and validity [27]. The Chinese version of the SSI was used in this study [28]. We set the SSI total score ≥ 6 as the cutoff for high levels of suicidal ideation, which has been shown to provide the best classification accuracy for predicting future suicidal behavior [29]. The Cronbach’s alpha in this study was 0.86.

The Suicidal Behaviors Questionnaire-Revised (SBQ-R) is a measure of the frequency and severity of past suicidal behaviors [30]. The SBQ-R includes four self-report items, each of which investigates a different dimension of suicidality. Higher total scores represent more severe suicidal behaviors. A cutoff score of SBQ-R ≥ 7 was used for identifying individuals at risk of suicidal behaviors and attempts [30]. The Chinese version of the SBQ-R was used in this study and had a Cronbach’s alpha of 0.79 [31]. The Cronbach’s alpha in this study was 0.81.

Demographic characteristics such as sex, age, education level, major, and school were also collected.

### 2.3. Statistical Analysis

Descriptive statistics (Mean [SD] and N [%]) were used to examine the characteristics of the sample. Pearson correlation analysis was applied to examine the interrelations between all key variables in the study using IBM SPSS Statistics (version 23.0). *p* values < 0.05 were considered to be statistically significant.

The SEM was used to explain the relationships and construct a structural equation model among stress, sleep, and suicide. The SEM was conducted using Amos (version 24.0) to test the path analysis, and it was constructed using the following steps: constructing theoretical models, testing for common method bias, performing confirmatory factor analysis, and testing for mediation effects on the model. The proposed mediating relationship was estimated by employing the maximum likelihood approach. Three criteria were used to evaluate the modeling fitness: (1) For the χ^2^/*df*, 1 < χ^2^/*df* < 3 suggests that the theoretical model is well fitted to the sample data (if the sample size is large, χ^2^/*df* = 5 is acceptable) [32]. (2) For the comparative fit index (CFI) and the normed fit index (NFI), both values above 0.90 indicated a good model fit [33]. (3) For the root mean square error of approximation (RMSEA), *RMSEA* < 0.05 is equal to a “close fit” (0.05 ≤ *RMSEA* < 0.08 is equal to an “acceptable fit”) [34]. The 95% bias-corrected bootstrap confidence interval (CI) and 95% percentile CI based on 3000 bootstrap samples were used for testing the mediating effects. All the indices and mediating effects were determined when zero was not included in the respective CI [35].

## 3. Results

### 3.1. Sample Characteristics

A total of 806 Chinese students were approached for the survey, and 780 responses were included in the analysis, corresponding to an effective response rate of 96.8%. The average age of the 780 participants was 20.58 years (SD = 2.57). Sample characteristics are summarized in Table 1.

The means and standard deviations for all of the key variables and the prevalence of childhood trauma, low sleep quality, and high suicide risk are shown in Table 2. In addition, the incidences of childhood trauma were calculated for participants whose SSI and/or SBQ scores exceeded the cutoff values.

### 3.2. Correlational Analysis

The bivariate correlations for all of the key variables in this study are displayed in Table 3. The results show that the subscale and total scores of CTQ and the PSS score correlated positively with PSQI, SSI, and SBQ (*p* < 0.05). Furthermore, the association between sleep and suicide is positively significant (*p* < 0.05). These findings offer a robust foundation for conducting the SEM analysis.

### 3.3. Mediation Analysis

**Figure 1:**
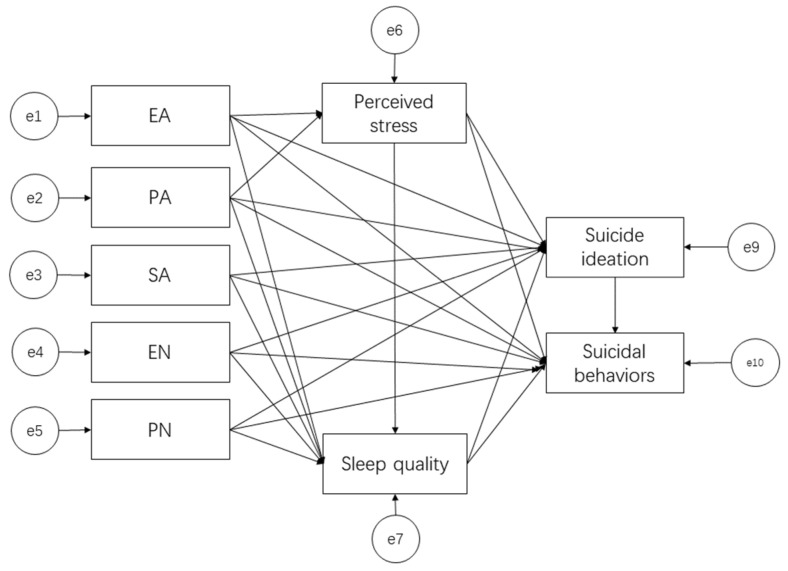
The theoretical model of mediation factors for suicidal ideation and behaviors (all assumed paths are positive predictive associations).

According to the results of the regression analysis, a theoretical model for the association between childhood trauma, perceived stress, sleep, and suicidal ideation and behavior was developed, as shown in Figure 1.

#### 3.3.1. Common Method Bias

A Harman’s Test showed that there was no common method bias (χ^2^ = 871.37, *df* =27, χ^2^/*df* = 32.27, *RMSEA* = 0.20, *CFI* = 0.65, *NFI* = 0.64).

#### 3.3.2. SEM

**Figure 2:**
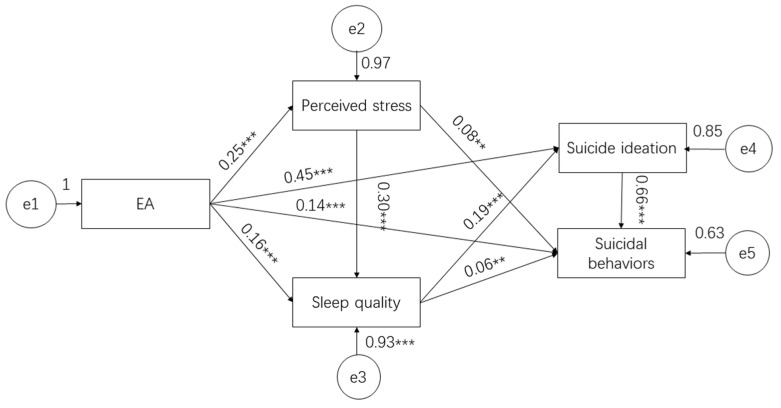
Standardized estimates for the structural model of childhood EA, perceived stress, and sleep quality on suicidal behavior. Significant paths are presented by asterisks (** *p* < 0.01, *** *p* < 0.001).

The fit indices in the theoretical equation model were not satisfactory (χ^2^= 1267.20, *df* = 18, χ^2^/*df* = 70.40, *RMSEA* = 0.30, *CFI* = 0.48, *NFI* = 0.48). Despite removing unreasonable paths in the model (e.g., that SA negatively predicts suicidal ideation) and modifying the model according to the modification indices, we attained a final equation model (Figure 2) with satisfactory fit indices (χ^2^ = 1.52, *df* = 1, χ^2^/*df* = 1.52, *RMSEA* = 0.03, *CFI* = 1.00, *NFI* = 1.00), which was acceptable (Figure 2).

The results of the hierarchical regression showed that all standardized path coefficients in this model were statistically significant: EA ^®^ perceived stress (β = 0.42, *p* < 0.001), EA ^®^ sleep quality (β = 0.16, *p* < 0.001), EA ^®^ suicidal ideation (β = 0.78, *p* < 0.001), EA ^®^ suicide behaviors (β = 0.13, *p* < 0.001), perceived stress ^®^ sleep quality (β = 0.19, *p* < 0.001), perceived stress ^®^ suicide behaviors (β = 0.13, *p* < 0.001), perceived stress ^®^ suicide behaviors (β = 0.04, *p* = 0.002), sleep quality ^®^ suicidal ideation (β = 0.32, *p* < 0.001), sleep quality ^®^ suicide behaviors (β = 0.06, *p* = 0.014), and suicidal ideation ^®^ suicide behaviors (β = 0.13, *p* < 0.001).

#### 3.3.3. Mediation Analysis

The mediating (indirect) effects between EA, perceived stress, sleep quality, suicidal ideation, and suicidal behaviors were tested using PROCESS. This method generates bootstrap CI for indirect effects of the predictors on the outcome variable through the mediator variables.

The hypothetical model included the following:

Model 1: EA ^®^ perceived stress ^®^ sleep quality ^®^ suicidal ideation ^®^ suicide behaviors;
Model 2: EA ^®^ perceived stress ^®^ sleep quality ^®^ suicide behaviors;
Model 3: EA ^®^ perceived stress ^®^ suicide behaviors;
Model 4: EA ^®^ sleep quality ^®^ suicidal ideation ^®^ suicide behaviors;
Model 5: EA ^®^ sleep quality ^®^ suicide behaviors;
Model 6: EA ^®^ suicidal ideation ^®^ suicide behaviors.

Table 4 shows that all the model pathways were significant. The total effect of EA on suicide behaviors was 49.5%. The mediating effects accounted for 73.7% of the total effects of EA on suicidal behaviors. Of all the mediating effects, effects through perceived stress and sleep quality accounted for 17.8%.

## 4. Discussion

This study aimed to examine the relationship between childhood trauma as an essential determinant of suicidal ideation and suicidal behavior. Furthermore, this study investigated the mediating role of perceived stress and sleep quality in the relationship. The results showed that childhood trauma, perceived stress, and sleep quality were all positively associated with suicidal ideation and suicidal behavior. However, in all types of childhood trauma, only EA could predict suicidal ideation and suicide behavior mediated by perceived stress and sleep quality. In addition, the total effects of EA on suicide behaviors were 49.5%, of which 17.8% was achieved through perceived stress and sleep quality.

The incidence of trauma in this study is similar to that of previous studies in China [20,36] and showed a moderately low level in the multi-country meta-analysis data [37]. The prevalence rates of all kinds of childhood trauma of the participants in this study were lower than those reported in previous studies with patients with depression, bipolar disorder, and schizophrenia [20,38]. Comparing data with a previous study conducted in Chinese university students, our results showed a greater proportion of participants with SSI or SBQ scores exceeding the cutoffs [39]. However, the proportion is far less than that of firefighters and psychiatric inpatient population [29]. The prevalence rates of childhood trauma in participants with SSI and/or SBQ scores exceeding the cutoffs were significantly higher than those of the overall participants

According to previous studies, childhood trauma experiences were strongly associated with higher suicide risks [40,41], which is consistent with our findings. Numerous studies have found an association between stress and sleep [7], between stress and suicide [8], and between sleep and suicide [9]. This study examined the mediating role of perceived stress and sleep quality between childhood trauma and suicide risk, which was an integration of these associations. It verified that experiencing childhood EA can increase the risk of suicide through perceived stress and poor sleep quality. At the same time, this study sheds light on the complex process of how childhood trauma increases suicide risk. Although this study only involved a small pathway, it is of interest for future research to uncover the complex processes that lead to suicide.

Furthermore, this study found that only EA could predict suicide risks through perceived stress and sleep quality. EA includes the restriction of movement; patterns of belittling, blaming, threatening, frightening, discriminating against, or ridiculing; and other non-physical forms of rejection or hostile treatment. It involves both isolated incidents as well as a pattern of the caregivers’ failure to provide a developmentally appropriate and supportive environment in a child’s growth [42]. The results indicate that different types of childhood trauma may create different pathways for suicide risks. Numerous studies verified that different types of childhood trauma may lead to varied outcomes. For example, EA was associated with the highest incidence rates of revictimization and PTSD symptom severity compared with other types of maltreatment [43]. EA, but not neglect, was correlated with an increased risk of psychosis and injecting-drug use [44]. Schizophrenia was related to higher EN and PN scores and a lower PA score [20]. It is worth noting that several studies found that EA is more strongly related to depression than SA and PA [20,45,46,47]. A study tried to explain the results by indicating that emotion dysregulation and interpersonal problems mediated the relationship between EA and depression [47]. Since emotional dysregulation, interpersonal problems, and depression are all risk factors of suicide [3], they may also be the mediating factors between EA and suicide risk and may explain why only EA could predict suicide risk in our model. However, further research is needed to verify this hypothesis. In addition, more research is also needed to differentiate between different categories of childhood trauma to reveal the complex process by which it increases suicide risk. In addition, further research could examine the pathways of different childhood traumatic experiences on suicide.

The results of this study provide recommendations for suicide prevention and intervention, particularly among individuals with experiences of childhood EA. Several psychotherapeutic approaches, such as cognitive-behavioral therapy (CBT) and dialectical behavior therapy (DBT), have been shown to not only have a positive effect on suicide risk but also improve sleep or stress. This may be one of the reasons why these methods work so well in preventing suicide. For example, a previous study found that CBT for insomnia not only improved sleep but also reduced levels of suicidal ideation [48]. Moreover, DBT helped reduce self-harm and suicide attempts in highly suicidal self-harming adolescents [49] while simultaneously lowering perceived stress [50]. However, few studies have verified whether a certain intervention is effective for stress, sleep, and suicide risks at the same time. A recent research study found that brief mindfulness meditation could help reduce suicidal ideation, stress, and sleep disturbance for individuals with high suicidal ideation [51]. Moreover, the study proposed that brief mindfulness meditation could reduce suicidal ideation not only directly but also indirectly by mediating stress, sleep, or the path of stress to sleep. However, the findings failed to verify this hypothesis. In addition to the relatively limited sample size, the results may have been due to the limited experiences of childhood EA among participants. In addition, the results of the current study suggest that crisis intervention workers should pay attention to the perceived stress and sleep problems of clients with childhood EA experiences. Psychotherapists may consider using the abovementioned psychotherapies or a combination of various psychotherapies simultaneously to reduce stress, sleep, and suicide risk and achieve better outcomes. Further empirical studies are needed to verify the impact of different psychotherapies on suicide risks and their risk factors simultaneously. Furthermore, it is recommended that when exploring the effects of suicide prevention and intervention, the impact of childhood traumatic experiences on outcomes be examined.

The following limitations of this study should be considered while interpreting our findings. First, the participants of this study were Chinese undergraduate and graduate students aged 18–30, which makes the findings most applicable to young Chinese people. Second, perceived stress and sleep quality data were collected through self-report questionnaires that largely depend on subjective feelings, which may have affected the objectivity and accuracy of the data. Third, the Cronbach’s alpha in this study was lower than 0.7. After double-checking, no missing data or unreasonable answers were found. The method of removing measures with more than three standard deviations of PSQI did not improve Cronbach’s alpha. Owing to the results of the *KMO* measure of sampling adequacy (*KMO* = 0.71, *p* < 0.001) and the experience of previous studies that acceptance reliability less than 0.7 instead of modifying the questionnaire, we used the primary measure [52]. Fourth, since the participants were not recruited from patients with clinical mental disorders, and the means of each variable were lower than the cut-offs, the results might be not necessarily applicable to clinical patients. Finally, this study was based on cross-sectional data. For future research, longitudinal and experimental research should be conducted to determine any causal relationships between relevant variables.

## 5. Conclusions

In this study, by constructing a structural equation model, it is verified that only EA, not PA, SA, EN, and PN, could predict suicide ideation and suicidal behavior mediated by perceived stress and sleep quality. This study is an attempt to explore the complex process of suicide formation, which hopefully could help to shed some light on how past traumas influences suicidal risks both directly and indirectly. In addition, the results indicate that different types of childhood trauma may create different pathways for suicide risks. It would be reasonable to invite clinical and mental health professionals to take into account how different types of traumatic experience clients had would influence their suicidal risks differently when conducting suicide assessment and intervention for individuals with childhood emotional abuse experiences. Furthermore, a proposal was made, based on the finding of this study, to consider potentially including additional focus of intervention on sleep and stress due to the mediation effects of stress and sleep.

## Figures and Tables

**Table 1:** Demographic characteristics of the study sample (*N* = 780).

Variables		*N*	%
Sex	Male	214	27.4
	Female	566	72.6
Education	Undergraduate	647	82.9
	Graduate	133	17.1
Major	Liberal Arts	208	39.5
	Science and Engineering	472	60.5
School	Key University	273	35
	General University	222	28.5
	Vocational college	285	36.5

**Table 2:** The means and standard deviations for all the key variables and the prevalence of childhood trauma, low sleep quality, and high suicide risk (*N* = 780).

Variables	M (SD)	Prevalence						
			All		SSI Total ≥ 6		SBQ Total ≥ 7	
		Cutoffs	*n*	%	*n*	%	*n*	%
CTQ EA	7.38 (2.97)	CTQ EA ≥ 13	60	7.7	44.0	73.3	46.0	76.7
CTQ PA	6.07 (2.08)	CTQ PA ≥ 10	47	6.0	33.0	70.2	27.0	57.4
CTQ SA	5.86 (2.03)	CTQ SA ≥ 8	95	7.9	42.0	44.2	36.0	37.9
CTQ EN	10.36 (4.69)	CTQ EN ≥ 15	135	17.3	79.0	58.5	65.0	48.1
CTQ PN	7.92 (2.82)	CTQ PN ≥ 10	199	25.5	90.0	45.2	73.0	36.7
CTQ Total	37.59 (10.89)							
PSS Total	19.60 (4.94)							
PSQI Total	6.63 (3.00)	PSQI Total > 7	281	36.0				
SSI Total	5.35 (5.11)	SSI Total ≥ 6	262	33.6				
SBQ Total	5.23 (2.88)	SBQ Total ≥ 7	206	26.4				

CTQ, Short Form of the Childhood Trauma Questionnaire; EA, emotional abuse; PA, physical abuse; SA, sexual abuse; EN, emotional neglect; PN, physical neglect; PSS, Perceived Stress Scale; PSQI, Pittsburgh Sleep Quality Index; SSI, Beck Scale for Suicide Ideation; SBQ-R, Suicidal Behaviors Questionnaire-Revised.

**Table 3:** Correlations of all the key variables (*N* = 780).

Variables	1	2	3	4	5	6	7	8	9
1 CTQ EA	1.00								
2 CTQ PA	0.60 ***								
3 CTQ SA	0.33 ***	0.39 ***							
4 CTQ EN	0.48 ***	0.33 ***	0.22 ***						
5 CTQ PN	0.45 ***	0.37 ***	0.31 ***	0.65 ***					
6 CTQ Total	0.77 ***	0.67 ***	0.53 ***	0.84 ***	0.79 ***				
7 PSS Total	0.25 ***	0.19 ***	0.07	−0.06	−0.01	0.09 *			
8 PSQI Total	0.23 ***	0.20 ***	0.10 **	0.18 ***	0.17 ***	0.24 ***	0.34 ***		
9 SSI Total	0.50 ***	0.35 ***	0.11 **	0.35 ***	0.28 ***	0.44 ***	0.21 ***	0.30 ***	
10 SBQ Total	0.50 ***	0.29 ***	0.14 ***	0.29 ***	0.22 ***	0.40 ***	0.27 ***	0.32 ***	0.76 ***

* *p* < 0.05, ** *p* < 0.01, *** *p* < 0.001; CTQ, Short Form of the Childhood Trauma Questionnaire; EA, emotional abuse; PA, physical abuse; SA, sexual abuse; EN, emotional neglect; PN, physical neglect; PSS, Perceived Stress Scale; PSQI, Pittsburgh Sleep Quality Index; SSI, Beck Scale for Suicide Ideation; SBQ-R, Suicidal Behaviors Questionnaire-Revised.

**Table 4:** The standardized meditating effects of EA on suicidal behaviors with perceived stress, sleep quality, and suicidal ideation as mediators (*N* = 780).

		Percentile 95%CI	
Model Pathways	The Standardized Meditating Effects	Lower	Upper
Model 1	0.25 × 0.30 × 0.19 × 0.66 = 0.01	0.003	0.022
Model 2	0.25 × 0.30 × 0.06 = 0.005	0.0003	0.013
Model 3	0.25 × 0.08 = 0.02	0.006	0.040
Model 4	0.16 × 0.19 × 0.66 = 0.02	0.005	0.045
Model 5	0.16 × 0.06 = 0.01	0.001	0.026
Model 6	0.45 × 0.66 = 0.30	0.218	0.382

Bootstrap replicates = 3000. EA, emotional abuse; CI, confidence interval.

## Data Availability

The datasets generated during the current study are not publicly available but are available from the corresponding author on reasonable request.
